# Effects of *Dichrostachys glomerata* and *Cissus quadrangularis* Extracts on GLP-1 Secretion and DPP-4 Activity in Overweight and Obese Individuals: A Randomized Controlled Trial

**DOI:** 10.3390/medicina62010041

**Published:** 2025-12-25

**Authors:** Janvier Youovop, Guy Takuissu, Régine Minoue, Felix Nwang, Maryam Adegboyega, Crista Arrey, Inelle Makamwe, Julius Oben

**Affiliations:** 1 Laboratory of Nutrition and Nutritional Biochemistry, Department of Biochemistry, University of Yaounde 1, Yaounde P.O. Box 812, Cameroon; j.youovop@jnaobenfoundation.org (J.Y.);; 2 Research and Development Department, Cameroon Nutrition and Dietetic Research Centre, J&A Oben Foundation, Yaounde P.O. Box 8348, Cameroonr.minoue@jnaobenfoundation.org (R.M.); m.adegboyega@jnaobenfoundation.org (M.A.); 3 Centre for Food, Food Security and Nutrition Research (CRASAN), Institute for Medical Research and Medicinal Plant Studies (IMPM), Ministry of Scientific Research and Innovation, Yaounde P.O. Box 13033, Cameroon; 4 National Obesity Centre, Central Hospital, Yaounde P.O. Box 87, Cameroon

**Keywords:** obesity, GLP-1, DPP-4, *Cissus quadrangularis*, *Dichrostachys glomerata*

## Abstract

*Background and Objectives*: *Dichrostachys glomerata* and *Cissus quadrangularis*, two species traditionally used in Cameroon, are recognized for their weight-reducing potential. This study examined the effects of standardized extracts of these botanicals on glucagon-like peptide-1 (GLP-1), dipeptidyl peptidase-4 (DPP-4), and key metabolic outcomes in individuals with excess body weight. *Materials and Methods*: In this 16-week, randomized, double-blind, placebo-controlled trial, 248 adults (126 women and 122 men; mean age 41.3 ± 0.3 years; BMI 25–34.9 kg/m^2^) were assigned to receive 400 mg *D. glomerata* extract (DGE), 300 mg *C. quadrangularis* extract (CQE), semaglutide (dose-escalated from 3 mg to 14 mg), or placebo, administered once daily. These are all standard clinical regimens. Primary assessments included changes in GLP-1 levels and DPP-4 activity. Secondary evaluations included body composition, caloric intake, satiety response, fasting glucose levels, and lipid profiles. *Results*: Participants receiving DGE or CQE displayed notable elevations in circulating GLP-1 (+38.6 pg/mL and +42.2 pg/mL, respectively; *p* < 0.01) and significant reductions in DPP-4 activity (−15.3% and −17.8%; *p* < 0.01) compared with placebo. Both extracts produced substantial improvements in body weight (−5.2% and −5.8%), body fat (−10.3% and −10.9%), energy intake (−16.2% and −17.5%), and satiety (+25.6% and +27.4%) (*p* < 0.01). Significant changes in fasting glucose and serum lipid levels were also observed (*p* < 0.05). These responses are comparable to those of semaglutide. Moreover, GLP-1 increments showed strong negative correlations with body fat percentage (r = −0.91 to −0.92; *p* < 0.001) and DPP-4 activity (r = −0.97 to −0.98; *p* < 0.001). *Conclusions*: Supplementation with *D. glomerata* and *C. quadrangularis* extracts enhanced GLP-1 secretion and reduced DPP-4 activity, yielding significant benefits for body composition and metabolic parameters. These findings indicate that both botanicals are promising natural agents for managing obesity through incretin-based mechanisms.

## 1. Introduction

Obesity and related disorders affect more than 2.3 billion individuals globally and contribute substantially to cardiometabolic morbidity [1]. Glucagon-like peptide-1 (GLP-1) is an important incretin hormone implicated in appetite regulation and weight loss [2]. Its physiological activity is limited by the enzyme dipeptidyl peptidase-4 (DPP-4), which rapidly degrades GLP-1 and thereby contributes to the development and progression of obesity and other metabolic diseases. Pharmacological agents such as DPP-4 inhibitors (gliptins, such as sitagliptin, saxagliptin, and vildagliptin) can enhance incretin signaling, but their use may be constrained by adverse effects and cost barriers [3]. Therefore, natural alternatives with fewer adverse effects are needed.

*Dichrostachys glomerata* and *Cissus quadrangularis*, traditionally used in Africa and Asia, promote weight loss; however, their underlying mechanisms remain unclear [4]. Clinical [5] and preclinical studies [6,7,8] suggest potential roles in DPP-4 inhibition, appetite suppression, and lipid metabolism. Despite these findings, evidence on GLP-1 and DPP-4 modulation in humans is lacking and constitutes an important gap in the field. This clinical trial evaluated the effects of these regimens on GLP-1, DPP-4, and other metabolic parameters in overweight and obese adults.

## 2. Materials and Methods

### 2.1. Test Materials

*Dichrostachys glomerata* extract (DGE) and *Cissus quadrangularis* extract (CQE), which are known for their anti-obesity effect, were supplied by Gateway Health Alliances Inc. (Fairfield, CA, USA). Each extract was standardized and accompanied by a certificate of analysis (COA) confirming identity, purity, and batch quality.

- DGE: Standardized to contain ≥25% total polyphenols, expressed as gallic acid equivalents (GAE).
- CQE: Standardized to contain ≥2.5% ketosteroids and ≥15% total flavonoids, expressed as quercetin equivalents (QE).

Both extracts were manufactured under Good Manufacturing Practice (GMP) conditions. Product characteristics are reported in Table 1.

Quality control and phytochemical verification were conducted at the Laboratory of Nutritional Biochemistry, University of Yaoundé I, using spectrophotometric assays for total phenolics and flavonoids. Chromatographic fingerprints were generated by HPLC-DAD to ensure batch uniformity and chemical consistency.

For comparison, oral semaglutide (Rybelsus^®^) was purchased commercially (Novo Nordisk A/S, Bagsværd, Gladsaxe Municipality, Denmark) and repackaged into visually matched capsules. Placebo capsules contained dextrin powder (400 mg) and were indistinguishable from the active capsules in size, color, and shape to preserve blinding.

### 2.2. Participants and Study Design

This randomized, double-blind, placebo-controlled, parallel-group clinical trial was registered at ClinicalTrials.gov (Identifier: NCT06827002) and conducted at the National Obesity Center of the Yaounde Central Hospital and the Laboratory of Nutrition and Nutritional Biochemistry, University of Yaoundé I. Ethical approval was granted by the Institutional Review Board (IRB) of the University of Yaoundé I (Approval code: BTC-JIRB2023-084) on 26 March 2023. Prior to Ethical Approval and to being within the required funding start date, study activities were limited to non-interventional, minimal-risk preparatory procedures, including site preparation and staff training. No participant enrollment, informed consent, randomization, intervention administration, or collection of outcome data occurred during this period. This study adhered to CONSORT 2010 and SPIRIT guidelines. Written informed consent was obtained from all the participants prior to enrolment, and the study complied with the principles of the Declaration of Helsinki (revised in 2013). Participant confidentiality and data privacy were maintained throughout the study.

This randomized, double-blind, placebo-controlled, parallel-group clinical trial was conducted at the National Obesity Center of the Yaounde Central Hospital and the Laboratory of Nutrition and Nutritional Biochemistry, University of Yaoundé I, and was subsequently registered at ClinicalTrials.gov (Identifier: NCT06827002).

The sample size (n) was calculated for the primary endpoint (change in postprandial GLP-1) using a two-sided, two-sample test for differences in means. Bonferroni correction was applied for the four planned pairwise comparisons (CQE vs. placebo, CQE vs. semaglutide, DGE vs. placebo, DGE vs. semaglutide) (α′ = 0.05/4 = 0.0125). The following formula was used:

where

n = required sample size per group;

α = nominal Type-I error (two-sided) with *Z*1 − α2 the corresponding normal quantile;

1 − β = desired power (80%), and *Z*1 − α2 was that quantile;

σ2 = variance; and

Δ = the minimum clinically meaningful difference between groups.

Allowing for a 15% dropout rate, the number of participants required per group increased to 62, yielding a total planned sample size of (n = 62 × 4) = 248.

A total of 248 overweight or obese adults (126 females and 122 males; age, 25–59 years) were recruited between March and September, 2023 through public advertisements and community health centers in Yaoundé, Cameroon.

### 2.3. Inclusion and Exclusion Criteria

➢ *Inclusion criteria*

- BMI between 25–34.9 kg/m^2^;
- Stable body weight (±2 kg) over the previous 3 months;
- Willingness to maintain usual dietary and physical activity patterns

➢ *Exclusion criteria*

- Diagnosed and uncontrolled diabetes mellitus;
- Recently suffered from a stroke or heart attack;
- Impaired kidney or liver function;
- Pregnancy or lactation;
- Use of weight-loss medications or herbal supplements within the previous 3 months;
- Known allergies to study materials.

### 2.4. Randomization

After screening, eligible participants were randomized (1:1:1:1) into four groups:

1. DGE 400 mg/day (dosage based on prior literature [9]);
2. CQE 300 mg/day (dosage based on prior literature [10]);
3. Oral semaglutide (dose-escalation: 3 → 7 → 14 mg/day);
4. Placebo (400 mg dextrin/day).

Randomization was performed by an IT technician not involved in the study using block randomization with a computer-generated random sequence (SPSS Version 26 random number generator). Allocation concealment was maintained using sequentially numbered, opaque, and sealed envelopes. Investigators and participants remained blinded to the group assignments.

### 2.5. Intervention Protocol

Participants consumed one capsule daily of one of the following: DGE, CQE, semaglutide or placebo for 16 consecutive weeks with water, 30 min before breakfast. Compliance was monitored through capsule counts at weeks 4, 8, 12, and 16, and through self-reported adherence logs. Missed doses were documented.

To help mitigate adverse gastrointestinal effects, semaglutide was administered following the dose-ascending protocol described by [11]. The starting dose of once-daily oral semaglutide was 3 mg (weeks 0–4), escalating to 7 mg (weeks 4–8), and then 14 mg (weeks 8–16).

### 2.6. Dietary and Exercise Restrictions

No specific dietary or exercise regimen was prescribed. Participants maintained their usual lifestyle patterns and refrained from initiating new nutritional supplements or medications that could affect their metabolism. Compliance with lifestyle maintenance was assessed through weekly self-reports and a review of 7-day food diaries.

### 2.7. Blood Sample Collection

Venous blood samples were collected at baseline (week 0) and at weeks 4, 8, 12 and 16 after an overnight fast and again 2 and 3 h postprandially. Samples were transferred into pre-chilled EDTA tubes with (PW) or without (PWO) sitagliptin. Plasma was separated by centrifugation (3000 rpm, 10 min, 4 °C) and stored at −80 °C until analysis.

### 2.8. Outcome Measures

#### 2.8.1. Primary Outcomes

- GLP-1 levels: PW was used to measure active GLP-1 in fasting as well as 2 and 3 h postprandial plasma. Concentrations were measured using the RayBio^®^ Human GLP-1 (Active) ELISA Kit (Catalog #ELH-GLP1-1, RayBiotech, Peachtree Corners, GA, USA).
- DPP-4 activity: PWO was assayed using a Human DPP-4/CD26 Immunoassay Kit (R&D Systems, Minneapolis, MN, USA) following the manufacturer’s instructions.

#### 2.8.2. Secondary Outcomes

- Anthropometric parameters: height (m) was measured using a stadiometer, weight (kg) using a digital scale (Omron HBF-511), BMI calculated as kg/m^2^, and body fat (%) estimated through bioelectric impedance (HD Touch Body composition scale).
- Metabolic parameters: Fasting glucose was measured with an Accu-Chek^®^ glucometer; total cholesterol, triglycerides, and HDL-c were measured using Roche Diagnostics kits (Mannheim, Germany); and LDL cholesterol levels were calculated using the Friedewald formula [12].
- Energy intake: Estimated from 7-day food diaries using the FAO Food Composition Tables for Cameroon. The energy intake (kcal/day) was calculated as follows:

EI=(4×[Carbohydrate+Protein]+9×Lipid)

- Satiety: Evaluated using the Visual Analogue Scale (VAS) questionnaire validated by Cazzo et al. [13].

### 2.9. Statistical Analysis

Data were analyzed using SPSS (version 24.0; IBM Corp., Chicago, IL, USA) and cross-validated using R version 4.3.1. Continuous variables are presented as mean ± standard error of the mean (SEM). The normality of data distributions was assessed using the Kolmogorov–Smirnov test as well as the visual inspection of Q–Q plots, and the homogeneity of variances was verified using Levene’s test. Outliers were identified as standardized residuals exceeding ±3 SD and evaluated for biological plausibility.

Between-group differences at baseline and at each time point were assessed using one-way analysis of variance (ANOVA) for normally distributed variables and the Kruskal–Wallis test for non-normal variables. Longitudinal changes were analyzed using repeated-measures ANOVA, with time (weeks 0, 4, 8, 12, and 16) as the within-subject factor and treatment group (placebo, DGE, and CQE) as the between-subject factor. Mauchly’s test assessed sphericity, and Greenhouse–Geisser corrections were applied when this assumption was violated. Post hoc pairwise comparisons were adjusted using the Benjamini–Hochberg false discovery rate (FDR) procedure.

Effect sizes were expressed as partial η^2^ (0.01 = small, 0.06 = medium, 0.14 = large) with corresponding 95% confidence intervals. Correlations among GLP-1, DPP-4 activity, body fat percentage, and energy intake were assessed using Pearson’s or Spearman’s correlation coefficients, as appropriate.

Predictors of the GLP-1 response and weight change were identified using multivariate linear regression analyses adjusted for baseline BMI, age, and sex. The model assumptions (linearity, homoscedasticity, multicollinearity, and normality of the residuals) were verified prior to interpretation. Standardized beta coefficients and adjusted R^2^ values were quantified for predictor contributions.

An a priori power analysis conducted in G*Power v3.1 indicated that a total sample size of 75 participants (25 per group) would provide 80% power to detect a medium effect size (f = 0.25) at α = 0.05 in a repeated-measures ANOVA with five time points. This sample size also provided adequate power to detect a minimum correlation coefficient of at least r = 0.30 with 95% confidence. All tests were two-tailed, and statistical significance was set at *p* ≤ 0.05.

## 3. Results

### 3.1. Participant Baseline Characteristics

**Figure 1:**
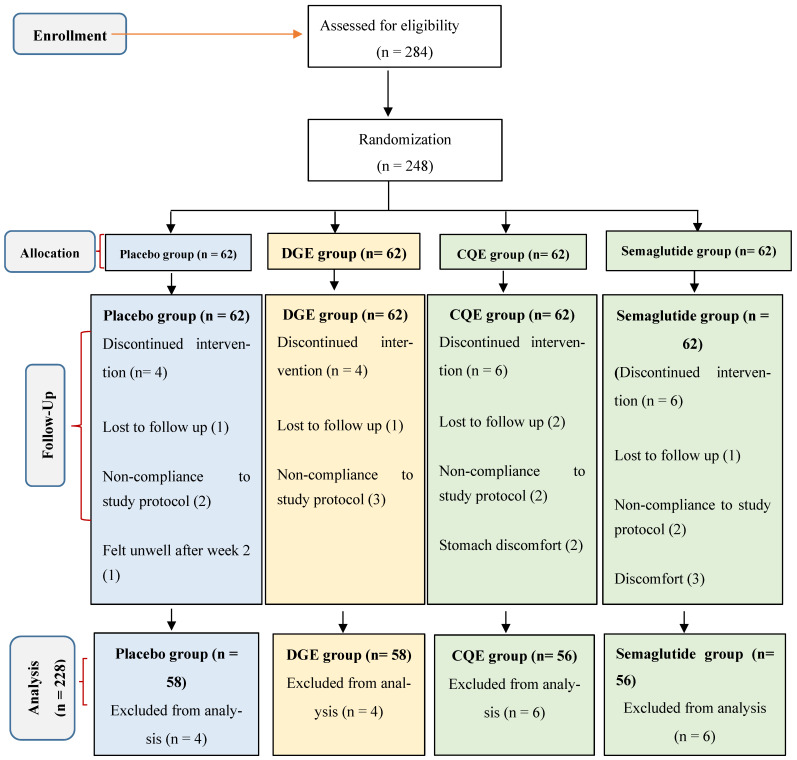
CONSORT flow diagram for clinical trial.

Among the 284 individuals screened, 248 participants (126 females and 122 males; mean age = 41.3 ± 0.55 years; mean BMI = 30.7 ± 2.4 kg/m^2^) met the inclusion criteria and were randomized equally into four groups: DGE (400 mg/day), CQE (300 mg/day), semaglutide (oral, 3–14 mg/day), or placebo (400 mg dextrin). A total of 228 participants (91.9%) completed the 16-week intervention (Figure 1). Withdrawals were as a result of loss to follow-up (n = 5), stomach and general discomfort (n = 6), and non-compliance with capsule intake (n = 9). The baseline demographic and metabolic characteristics did not differ significantly across groups (Table 2).

### 3.2. Effects on Circulating GLP-1 and DPP-4 Activity

Postprandial GLP-1 concentrations increased significantly from baseline in all active treatment groups compared with placebo (time × treatment interaction, *p* < 0.001). At week 16, mean GLP-1 levels at three hours postprandial rose by +38.6 pg/mL in the DGE group, +42.2 pg/mL in the CQE group, and +46.8 pg/mL in the semaglutide group, compared with +4.7 pg/mL in the placebo group (Table 3). Similar patterns were observed at two hours postprandial. Post hoc Tukey tests confirmed significant differences between the DGE/CQEs and placebo groups (*p* < 0.01), while no significant differences were detected between the botanical groups and semaglutide (*p* > 0.05).

DPP-4 enzymatic activity decreased significantly in the DGE (−15.3%) and CQE (−17.8%) groups relative to placebo (−2.9%, *p* < 0.001), with the semaglutide group exhibiting the highest reduction (−23.5%) (Table 3).

### 3.3. Anthropometric and Metabolic Parameters

#### 3.3.1. Anthropometric Parameters

Body weight decreased significantly after 16 weeks in the DGE (−4.3 kg) and CQE (−4.7 kg) and semaglutide (−4.8 kg) groups compared with placebo (−0.7 kg, *p* < 0.05). Correspondingly, BMI and body fat percentage declined significantly in all active treatment groups (*p* > 0.05) (Table 4).

#### 3.3.2. Metabolic Parameters

Fasting glucose and triglyceride levels decreased significantly in all active treatment groups relative to placebo group (Table 5). LDL-C levels exhibited modest reductions and HDL-C levels modest increases in the DGE and CQE groups; however, these changes were not statistically significant after adjustment for multiple comparisons (adjusted *p* > 0.05) (Table 5).

### 3.4. Energy Intake and Satiety Scores

Daily energy intake declined significantly from baseline values in all groups, but with larger reductions observed in the DGE (−470.2 kcal/day) and CQE (−513.8 kcal/day) groups compared with placebo (−92 kcal/day, *p* < 0.05). VAS-based satiety scores increased by +25.6% in the DGE group and +27.4% in the CQE groups, compared with +5.3% in the placebo group (*p* < 0.01) (Table 6). Exploratory analysis indicated positive correlations between changes in satiety and GLP-1 levels. GLP-1 levels were negatively correlated with DPP-4 activity (Table 7).

### 3.5. Safety and Tolerability

No serious adverse events were observed. Mild gastrointestinal discomfort occurred primarily at the beginning of the intervention in the semaglutide group and in less than 3% of participants in the DGE and CQE groups. All events resolved without requiring discontinuation.

## 4. Discussion

This randomized controlled trial demonstrated that supplementation with DGEs and CQEs significantly enhanced the incretin axis in overweight and obese adults, as evidenced by increased circulating GLP-1 concentrations and reduced DPP-4 activity. These endocrine effects translated into clinically meaningful improvements, including reductions in body weight, body fat, energy intake, as well as enhanced satiety and favorable changes in lipid profile and fasting blood glucose. Collectively, these findings extend prior in vitro and preclinical evidence indicating that polyphenol-rich extracts modulate incretin signaling [14].

The dual mechanism of stimulating GLP-1 secretion and inhibiting its enzymatic degradation through DPP-4 suppression is characteristic of polyphenol-rich plant extracts. The flavonoid glycosides and related phytochemicals in DGE and CQE likely act as allosteric or competitive inhibitors of DPP-4, thereby prolonging the half-life of endogenous GLP-1 [15]. These actions operate in parallel, although at a nutraceutical scale, to the pharmacodynamics of synthetic GLP-1 receptor agonists and DPP-4 inhibitors such as semaglutide and sitagliptin. The degree of GLP-1 elevation observed in this trial suggests partial engagement of comparable signaling pathways. Moreover, accumulating evidence indicates that CQE polyphenols may directly stimulate GLP-1 secretion from intestinal L-cells through AMPK activation and intracellular calcium mobilization [16,17].

The reductions in body weight and adiposity likely reflected both incretin-mediated appetite regulation and the direct metabolic effects of these extracts. GLP-1 enhances satiety through hypothalamic signaling, which aligns with the increased VAS satiety scores and decreased energy intake observed in this study. CQE constituents also exert peripheral antiadipogenic and lipolytic effects. For instance, a lupenone-rich CQE fraction suppressed lipid accumulation in 3T3-L1 adipocytes [5], while a human trial demonstrated CQE-induced upregulation of uncoupling protein (UCP) mRNA, indicating increased white adipose tissue browning [18]. Browning enhances energy expenditure and fat oxidation, thereby contributing to reductions in central adiposity.

These metabolic benefits may be further potentiated by the anti-inflammatory properties of these extracts. *Cissus quadrangularis* inhibits key pro-inflammatory pathways through specific pharmacophores [19]. By attenuating chronic low-grade inflammation mediated by cytokines such as TNF-α and IL-6—drivers of insulin resistance and adipose dysfunction—CQE fosters a metabolically favorable environment. This anti-inflammatory shift enhances insulin signaling and promotes the transition of the adipose tissue toward oxidative rather than storage functions, thereby reinforcing improvements in both body composition and glucose homeostasis.

The improvements in lipid profile and reductions in total cholesterol, triglycerides, and LDL-C align with known roles of GLP-1 in lipid metabolism, including the inhibition of hepatic lipogenesis and stimulation of lipoprotein lipase activity. Similar lipid-lowering effects have been reported in other clinical trials evaluating weight-loss supplementation [20]. The moderate but significant reduction in fasting glucose can be attributed to the combined effects of enhanced insulin secretion through GLP-1 signaling, improved insulin sensitivity associated with weight loss, and the β-cell protective properties of CQE [17].

Additionally, reductions in fasting glucose may result from the direct inhibition of carbohydrate-digesting enzymes. CQE was recently identified as a potent inhibitor of α-amylase and α-glucosidase [17]. Inhibiting these enzymes delays carbohydrate digestion and glucose absorption, reduces postprandial glycemic excursions, and lowers overall glycemic load. This mechanism complements incretin-mediated insulin enhancement, providing a multifaceted phytotherapeutic approach to glycemic control.

These findings are consistent with prior clinical trials demonstrating the anti-obesity and metabolic effects of *Cissus quadrangularis* [10,20] and *Dichrostachys glomerata* [4,15]. The magnitude of weight loss and metabolic improvement observed is consistent with prior reports, reinforcing the reproducibility of these effects. However, variations in extract standardization, duration, and dosage underscore the need for uniform formulations to optimize translational outcomes.

Despite these promising findings, this study has several limitations. The most significant of these is the asymmetric dosing protocols. We compared fixed doses of herbal extracts to a titrated, maximally effective dose of semaglutide. This design, while pragmatic, prevents a direct pharmacological comparison of the intrinsic efficacy or potency of the compounds. It cannot determine whether the tested doses of DGE or CQE represent their optimal efficacy (D_max), nor can it establish milligram-for-milligram equivalence. Comparability claims pertain specifically to the tested regimens, not to the agents in principle. Also, the 16-week duration restricts inferences regarding long-term adaptations to sustained GLP-1 elevation. Additionally, the study included overweight but nondiabetic adults, limiting generalizability to individuals with diabetes or normal BMI. Future studies should incorporate mechanistic endpoints, such as adipocyte gene expression, insulin sensitivity indices, and AMPK activation assays, to delineate the molecular pathways linking incretin modulation to metabolic outcomes.

## 5. Conclusions

In this 16-week trial, a fixed daily dose of DGE (400 mg) or CQE (300 mg) enhanced GLP-1 bioavailability and signaling, leading to significant improvements in body composition, appetite regulation, and metabolic health comparable to the standard clinical titration regimen of oral semaglutide (3 → 7 → 14 mg) as measured at the study endpoints. Beyond incretin modulation, these extracts may directly influence adipose tissue browning, lipid oxidation, and anti-inflammatory activity, offering a comprehensive nutraceutical approach to obesity management. Their multi-targeted biochemical effects warrant long-term, mechanistic studies to establish standardized and clinically validated formulations.

## Figures and Tables

**Table 1:** Test Product Details.

Extract	Batch/Lot No	Standardization Marker	Certificate of Analysis	Formulation	Supplier
DGE	GHA-DGE-0423	Polyphenols (25% GAE)	COA #DGE0423-24	Capsule, 400 mg	Gateway Health Alliances
CQE	GHA-CQE-0323	Ketosterols (2.5%) Flavonoids (15% QE)	COA #CQE0323-24	Capsule, 300 mg	Gateway Health Alliances

DGE: *Dichrostachys glomerata* extract; CQE: *Cissus quadrangularis* extract; GHA: Gateway Health Alliances; COA: certificate of analysis; QE: quercetin equivalents; GAE: gallic acid equivalents.

**Table 2:** Demographic and baseline characteristics of participants.

	Placebo (n = 58)	DGE (n = 58)	CQE (n = 56)	Semaglutide (n = 56)
Demographic Characteristics				
Age (years)	43.04 ± 1.05	41.37 ± 1.14	40.70 ± 1.12	40.09 ± 1.04
Gender (n)				
Males (%)	50.00 (29)	51.72 (30)	44.64 (25)	50.00 (28)
Females (%)	50.00 (29)	48.28 (28)	55.36 (31)	50.00 (28)
Primary Outcomes				
GLP-1 level (pg/mL)	13.00 ± 0.73	13.10 ± 0.65	13.13 ± 0.56	13.04 ± 0.34
DPP-4 activity (U/L)	31.68 ± 0.96	31.82 ± 1.14	31.42 ± 1.36	31.60 ± 0.96
Secondary Outcomes				
Anthropometric Characteristics				
Body Weight (kg)	81.97 ± 10.09	81.96 ± 9.48	81.55 ± 9.49	81.80 ± 1.22
Body Mass Index (kg/m^2^)	30.62 ± 1.34	30.68 ± 1.10	30.93 ± 1.58	30.56 ± 1.44
Body Fat Percentage (%)	30.94 ± 1.79	30.93 ± 1.86	31.26 ± 2.09	31.57 ± 2.56
Energy Intake & Satiety				
Energy intake (Kcal/day)	2943.93 ± 859.18	2898.60 ± 839.75	2941.64 ± 918.33	2940.60 ± 885.99
Satiety Score (VAS score 0–10)	8.20 ± 0.60	8.00 ± 0.40	8.10 ± 0.50	8.00 ± 0.32
Metabolic Characteristics				
Total Cholesterol (mg/dL)	191.72 ± 3.09	191.52 ± 2.39	190.88 ± 2.78	190.80 ± 2.23
Triglyceride (mg/dL)	58.78 ± 2.31	58.21 ± 2.46	58.71 ± 1.53	58.20 ± 2.07
LDL-c (mg/dL)	166.38 ± 3.16	167.65 ± 2.54	166.93 ± 2.86	165.90 ± 2.76
HDL-c (mg/dL)	66.89 ± 1.11	67.07 ± 1.74	66.71 ± 1.91	66.80 ± 1.60
Fasting blood glucose (mg/dL)	106.41 ± 3.93	106.33 ± 3.65	106.51 ± 3.33	106.40 ± 3.65

DGE: *Dichrostachys glomerata* extract; CQE: *Cissus quadrangularis* extract; DPP4: Dipeptidyl Peptidase 4; GLP-1: Glucagon-Like Peptide 1; LDL-c: Low-Density Lipoprotein Cholesterol; HDL-c: High-Density Lipoprotein Cholesterol.

**Table 3:** The effect of DGE and CQE on GLP-1 level and DPP4 Activity.

Groups	Baseline	Week 4	Week 8	Week 12	Week 16	Change over 16 Weeks
GLP-1 (pg/mL)						
Placebo	13.00 ± 0.73	13.58 ± 0.65	16.43 ± 0.64	17.48 ± 0.58	17.70 ± 0.67	4.70 (36.12%)
DGE	13.10 ± 0.65	21.92 ± 5.53 ^δ#^	31.57 ± 0.63 ^δ^*^#^*	44.90 ± 1.19 ^δ^	51.7 ± 1.02 ^δ^	38.6 (294.7%) ^δ^
CQE	13.13 ± 0.56	22.56 ± 0.88 ^δ^	34.15 ± 1.12 ^δ^	45.56 ± 1.11 ^δ^	55.33 ± 1.20 ^δ^	42.2 (321.4%) ^δ^
Semaglutide	13.04 ± 0.34	27.52 ± 3.12	45.28 ± 0.82	53.65 ± 1.24 ^δ^	59.84 ± 1.28 ^δ^	46.80 (358.9%) ^δ^
DPP4 (U/L)						
Placebo	31.68 ± 0.96	31.40 ± 2.15	31.10 ± 8.93	31.20 ± 8.05	30.90 ± 8.37	−0.78 (−2.9%)
DGE	31.82 ± 1.14	31.54 ± 2.77 ^δ#^	28.91 ± 3.16 ^δ^	28.32 ± 3.12 ^δ^	27.71 ± 2.93 ^δ^	−4.11 (−15.3%) ^δ^
CQE	31.42 ± 1.36	30.01 ± 1.90 ^δ^	28.27 ± 3.17 ^δ^	27.50 ± 2.84 ^δ^	26.72 ± 3.80 ^δ^	−4.70 (−17.8%) ^δ^
Semaglutide	31.60 ± 0.96	28.95 ± 1.74 ^δ^	27.34 ± 1.60 ^δ^	26.38 ± 2.68 ^δ^	25.34 ± 3.18 ^δ^	−6.26 (−23.5%) ^δ^

DGE: *Dichrostachys glomerata* extract; CQE: *Cissus quadrangularis* extract; DPP4: Dipeptidyl Peptidase 4; GLP-1: Glucagon-Like Peptide 1; Intergroup analysis: ^#^ *p* < 0.05: significantly different compared with the CQE in the same column; ^δ^ *p* < 0.01: significantly different from the placebo group in the same column; The last column represents changes compared to baseline over 16 weeks; the values in brackets represent the percentage of change over this period.

**Table 4:** The effect of DGE and CQE on anthropometric parameters.

Parameters	Groups	Baseline	Week 4	Week 8	Week 12	Week 16	Change over 16 Weeks
Body Weight (kg)	Placebo	81.97 ± 10.09	81.74 ± 9.80	81.52 ± 9.86	81.42 ± 10.87	81.31 ± 9.78	−0.60 (−0.7%)
	DGE	81.96 ± 9.48	81.22 ± 12.00	80.45 ± 13.12	79.86 ± 14.67 ^δ^	77.66 ± 12.52 ^δ^	−4.3 (−5.2%) ^δ^
	CQE	81.55 ± 9.49	80.92 ± 10.62	80.30 ±11.75	79.15 ± 12.84 ^δ^	76.85 ± 10.48 ^δ^	−4.7 (−5.8%) ^δ^
	Semaglutide	81.80 ± 1.22	80.18 ± 9.42	78.55 ± 9.92	77.58 ± 12.77 ^δ^	77.00 ± 10.78 ^δ^	−4.8 (−5.9%) ^δ^
Body Mass Index (kg/m^2^)	Placebo	30.62 ± 1.34	30.58 ± 1.36	30.50 ± 1.38	30.45 ± 1.24	30.40 ± 1.42	−0.22 (−0.7%)
	DGE	30.68 ± 1.10	30.44 ± 1.83 *^#^*	30.20 ± 1.68 ^δ^	29.93 ± 2.68 ^δ^	29.10 ± 1.82 ^δ^	−1.58 (−5.1%) ^δ^
	CQE	30.93 ± 1.58	30.11 ± 1.52 *	29.86 ± 1.83 ^δ^	29.62 ± 1.52 ^δ^	29.02 ± 2.10 ^δ^	−1.91 (−6.2%) ^δ^
	Semaglutide	30.56 ± 1.44	30.00 ± 1.28	29.41 ± 2.70 ^δ^	28.65 ± 0.96 ^δ^	28.44 ± 1.02 ^δ^	−2.12 (−6.9%) ^δ^
Body Fat Percentage (%)	Placebo	30.94 ± 1.79	30.97 ± 1.14	30.46 ± 0.71	30.48 ± 1.37	30.49 ± 1.73	−0.45 (−1.5%)
	DGE	30.93 ± 1.86	30.42 ± 1.47	29.19 ± 4.49 *	29.35 ± 6.86 *	27.73 ± 4.42 ^δ^	−3.2 (−10.3%) ^δ^
	CQE	31.26 ± 2.09	30.86 ± 3.23	30.46 ± 4.18	29.46 ± 3.31 ^δ^	27.86 ± 3.14 ^δ^	−3.4 (−10.9%) ^δ^
	Semaglutide	31.57 ± 2.56	30.37 ± 4.73 *	29.17 ± 4.89 *	28.58 ± 4.74 ^δ^	27.97 ± 3.47 ^δ^	−3.60 (−11.4%) ^δ^

DGE: *Dichrostachys glomerata* extract; CQE: *Cissus quadrangularis* extract; VAS: visual analogue scale; Intergroup analysis: * *p* < 0.05: significantly different from the placebo group in the same column; ^#^ *p* < 0.05: significantly different compared with the CQE in the same column; ^δ^ *p* < 0.01: significantly different from the placebo group in the same column. The last column represents changes compared to baseline over 16 weeks; the values in brackets represent the percentage of change over this period.

**Table 5:** The effect of DGE and CQE on metabolic parameters.

Parameters	Groups	Baseline	Week 4	Week 8	Week 12	Week 16	Change over 16 Weeks
Total cholesterol (TC)	Placebo	191.72 ± 3.09	191.45 ± 3.00	191.20 ± 3.21	191.09 ± 3.50	190.98 ± 3.78	−0.74 (−0.4%)
	DGE	191.52 ± 2.39	189.85 ± 2.60 ^δ*#*^	187.45 ± 2.84 ^δ*#*^	185.12 ± 4.02 ^δ*#*^	181.40 ± 3.50 ^δ^	−10.12 (−5.28%) ^δ^
	CQE	190.88 ± 2.78	187.12 ± 5.05 ^δ^	183.56 ± 4.73 ^δ^	179.45 ± 4.99 ^δ^	176.90 ± 4.20 ^δ^	−13.98 (−7.32%) ^δ^
	Semaglutide	190.80 ± 2.23	185.25 ± 4.82 ^δ^	179.90 ± 2.37 ^δ^	177.23 ± 2.84 ^δ^	174.55 ± 3.30 ^δ^	−16.25 (−8.5%) ^δ^
Triglycerides (TG)	Placebo	58.78 ± 2.31	58.65 ± 2.60	58.50 ± 2.05	58.46 ± 2.38	58.40 ± 2.68	−0.38 (−0.6%)
	DGE	58.21 ± 2.46	57.35 ± 2.68 ^δ^	56.10 ± 0.94 ^δ*#*^	55.05 ± 2.76 ^δ*#*^	53.30 ± 2.12 ^δ^	−4.9 (−8.42%) ^δ^
	CQE	58.71 ± 1.53	57.10 ± 1.66 ^δ^	55.65 ± 1.37 ^δ^	54.25 ± 1.81 ^δ^	52.50 ± 1.40 ^δ^	−6.21 (−10.58%) ^δ^
	Semaglutide	58.20 ± 2.07	55.95 ± 1.58 ^δ^	53.85 ± 1.10 ^δ^	52.65 ± 1.24 ^δ^	51.45 ± 1.78 ^δ^	−6.75 (−11.60%) ^δ^
LDL-c	Placebo	166.38 ± 3.16	166.20 ± 3.12	166.05 ± 3.07	165.95 ± 3.46	165.85 ± 3.86	−0.53 (−0.3%)
	DGE	167.65 ± 2.54	165.90 ± 2.76 ^δ*#*^	163.85 ± 2.92 ^δ*#*^	161.75 ± 4.18 ^δ*#*^	159.80 ± 3.08 ^δ^	−7.85 (−4.68%) ^δ^
	CQE	166.93 ± 2.86	163.55 ± 4.97 ^δ^	160.35 ± 4.65 ^δ^	157.10 ± 5.99 ^δ^	155.15 ± 5.02 ^δ^	−11.78 (−7.06%) ^δ^
	Semaglutide	165.90 ± 2.76	161.45 ± 3.15 ^δ^	157.10 ± 2.92 ^δ^	154.74 ± 2.84 ^δ^	152.35 ± 4.73 ^δ^	−13.55 (−8.2%) ^δ^
HDL-c	Placebo	66.89 ± 1.11	66.95 ± 0.94	67.00 ± 0.55	67.03 ± 0.72 ^δ^	67.05 ± 0.87	+0.16 (+0.2%)
	DGE	67.07 ± 1.74	67.45 ± 1.02 ^δ*#*^	67.80 ± 0.71 ^δ*#*^	68.15 ± 1.18 ^δ*#*^	68.50 ± 1.02 ^δ^	+1.43 (+2.13%) ^δ^
	CQE	66.71 ± 1.91	67.25 ± 3.00 ^δ^	67.75 ± 2.21 ^δ^	68.30 ± 2.76 ^δ^	68.75 ± 2.01 ^δ^	+2.04 (+3.06%) ^δ^
	Semaglutide	66.80 ± 1.60	67.50 ± 1.73 ^δ^	68.25 ± 0.94 ^δ^	68.68 ± 0.86 ^δ^	69.05 ± 0.79 ^δ^	+2.25 (+3.4%) ^δ^
Fasting Blood Glucose	Placebo	106.41 ± 3.93	106.35 ± 3.94	106.25 ± 3.39	106.20 ± 3.47	106.15 ± 3.55	−0.26 (−0.2%)
	DGE	106.33 ± 3.95	104.85 ± 4.86 ^δ*#*^	102.95 ± 5.76 ^δ*#*^	101.25 ± 6.24 ^δ*#*^	97.02 ± 5.55 ^δ^	−9.31 (−8.8%) ^δ^
	CQE	106.51 ± 3.33	104.10 ± 4.10 ^δ^	101.85 ± 3.16 ^δ^	99.45 ± 2.25 ^δ^	96.21 ± 2.70 ^δ^	−10.30 (−9.7%) ^δ^
	Semaglutide	106.40 ± 3.65	102.95 ± 3.94 ^δ^	99.25 ± 3.32 ^δ^	97.30 ± 3.16 ^δ^	95.35 ± 2.18 ^δ^	−11.05 (−10.4%) ^δ^

DGE: *Dichrostachys glomerata* extract; CQE: *Cissus quadrangularis* extract; LDL-c: Low-Density Lipoprotein Cholesterol; HDL-c: High-Density Lipoprotein Cholesterol; Intergroup analysis: ^#^ *p* < 0.05: significantly different compared with the CQE in the same column; ^δ^ *p* < 0.01: significantly different from the placebo group in the same column. The last column represents changes compared to baseline over 16 weeks; the values in brackets represent the percentage of change over this period.

**Table 6:** The effect of DGE and CQE on energy intake and Satiety.

Parameters	Groups	Baseline	Week 4	Week 8	Week 12	Week 16	Change over 16 Weeks
Energy Intake (Kcal/day)	Placebo	2943.93 ± 859.18	2921.39 ± 858.67	2818.39 ± 832.75	2835.16 ± 845.71	2851.93 ± 794.39	−92.0 (−3.1%)
	DGE	2898.60 ± 839.75	2891.06 ± 839.73	2704.06 ± 839.40 *	2660.60 ± 847.92 *	2428.40 ± 843.83 *	−470.20 (−16.2%) ^δ^
	CQE	2941.64 ± 918.33	2856.46 ± 927.41	2856.46 ± 933.48 *	2665.64 ± 942.98 *	2427.84 ± 902.42 *	−513.8 (−17.5%) ^δ^
	Semaglutide	2940.60 ± 885.99	2757.72 ± 852.78	2537.00 ± 931.12 *	2463.50 ± 891.95 *	2390.00 ± 866.76 *	−550.0 (18.7%) ^δ^
Satiety Score (VAS score 0–10)	Placebo	8.20 ± 0.60	8.24 ± 0.80	8.36± 0.60	8.61 ± 0.75	8.85 ± 0.70	0.65 (5.3%)
	DGE	8.00 ± 0.40	8.14 ± 0.70 ^δ^	8.38 ± 0.60 ^δ^	9.24 ± 0.40 ^δ^	10.05 ± 0.80 ^δ^	2.05 (25.6%) ^δ^
	CQE	8.10 ± 0.50	8.05 ± 0.60 ^δ^	8.90 ± 0.70 ^δ^	9.51 ± 0.60 ^δ^	10.32 ± 0.52 ^δ^	2.22 (27.4%) ^δ^
	Semaglutide	8.00 ± 0.32	9.72 ± 0.52 ^δ^	10. 45 ± 0.62 ^δ^	10.63 ± 0.80	10.77 ± 0.42 ^δ^	2.77 (34.6%) ^δ^

DGE: *Dichrostachys glomerata* extract; CQE: *Cissus quadrangularis* extract; VAS: visual analogue scale; Intergroup analysis: * *p* < 0.05: significantly different from the placebo group in the same column, ^δ^ *p* < 0.01: significantly different from the placebo group in the same column. The last column represents changes compared to baseline over 16 weeks; the values in brackets represent the percentage of change over this period.

**Table 7:** Correlation matrix between GLP-1, DPP-4, body fat, and energy intake in treated groups at week 16.

Parameters	GLP-1	DPP-4	Body Fat %	Energy Intake	VAS Score
DGE					
GLP-1	1	−0.82 *	−0.74 *	−0.75 *	0.84 *
DPP-4	−0.82 *	1	0.70 *	0.70 *	−0.79 *
Body fat	−0.74 *	0.70 *	1	0.63 *	−0.67 *
Energy intake	−0.75 *	0.70 *	0.63 *	1	−0.82 *
VAS score	0.84 *	−0.79 *	−0.67 *	−0.82 *	1
CQE					
GLP-1	1	−0.86 *	−0.78 *	−0.80 *	0.88 *
DPP-4	−0.86 *	1	0.74 *	0.76 *	−0.84 *
Body fat	−0.78 *	0.74 *	1	0.70 *	−0.73 *
Energy intake	−0.80 *	0.76 *	0.70 *	1	−0.86 *
VAS score	0.88 *	−0.84 *	−0.73 *	−0.86 *	1

* *p* < 0.001; DGE: *Dichrostachys glomerata* extract; CQE: *Cissus quadrangularis* extract; DPP-4: Dipeptidyl Peptidase 4; GLP-1: Glucagon-Like Peptide 1.

## Data Availability

The data utilized in this study is available upon reasonable request from the corresponding author.

## Abbreviations

The following abbreviations are used in this manuscript:

BMI	Body Mass Index
DPP4	Dipeptidyl Peptidase 4
GLP-1	Glucagon-Like Peptide-1
